# Rank aggregation of independent genetic screen results highlights new strategies for adoptive cellular transfer therapy of cancer

**DOI:** 10.3389/fimmu.2023.1235131

**Published:** 2023-12-08

**Authors:** Vianca V. Vianzon, Rylee M. Hanson, Ishita Garg, Gwenyth J. Joseph, Laura M. Rogers

**Affiliations:** Department of Immunology, Mayo Clinic, Rochester, MN, United States

**Keywords:** cancer immunotherapy, T cell trafficking, CAR-T, adoptive transfer therapy, Sleeping Beauty

## Abstract

Efficient intratumoral infiltration of adoptively transferred cells is a significant barrier to effectively treating solid tumors with adoptive cellular transfer (ACT) therapies. Our recent forward genetic, whole-genome screen identified T cell-intrinsic gene candidates that may improve tumor infiltration of T cells. Here, results are combined with five independent genetic screens using rank aggregation to improve rigor. This resulted in a combined total of 1,523 candidate genes – including 1,464 genes not currently being evaluated as therapeutic targets - that may improve tumor infiltration of T cells. Gene set enrichment analysis of a published human dataset shows that these gene candidates are differentially expressed in tumor infiltrating compared to circulating T cells, supporting translational potential. Importantly, adoptive transfer of T cells overexpressing gain-of-function candidates (*AAK1^ΔN125^*, *SPRR1B*, and *EHHADH*) into tumor-bearing mice resulted in increased T cell infiltration into tumors. These novel gene candidates may be considered as potential therapeutic candidates that can aid adoptive cellular therapy in improving T cell infiltration into solid tumors.

## Introduction

Adoptive cellular therapies (ACT) have emerged as a promising treatment for cancer, with some patients exhibiting durable clinical responses. T cell ACT is the autologous or allogeneic transplant of tumor infiltrating lymphocytes, T cell receptor (TCR) engineered, or chimeric antigen receptor (CAR) expressing T cells (1). ACTs encompass the largest number of agents in current development for immunotherapy of cancer (2). While most cancers in humans are solid tumors (~90%), less than half of all ACT clinical trials are directed toward solid tumors with a bias toward specific cancer types including melanoma, central nervous system malignancies, and liver cancer (2). This is due to unique challenges of targeting solid tumors that often have an immunosuppressive microenvironment and an extracellular matrix that excludes T cells.

T cell infiltration is critical for ACT success and current efforts are focusing on improving intratumoral T cell accumulation using approaches that include radiotherapy and oncolytic viruses to remodel the tumor microenvironment (3). However, T cell intrinsic mechanisms may represent attractive therapeutic targets, given that many ACT products are genetically manipulated *ex vivo* before being administered to the patient and providing the opportunity to genetically improve trafficking capabilities. We recently performed a whole genome Sleeping Beauty (*SB*) screen in T cells and identified 856 (FDR < 0.01) gene candidates that may improve intratumoral accumulation of adoptively transferred T cells (4). These candidates encode proteins with diverse functional annotations that may support T cell priming, proliferation, migration, and persistence in the tumor microenvironment.

Here, we combine our screen results with those from several independent genetic screens in T cells to assess the selected candidates more rigorously. This was accomplished through two complimentary approaches: simple overlap analysis and formal rank aggregation. Using gene set enrichment, we demonstrate that the T cell gene candidates are differentially expressed in tumor infiltrating versus circulating T cells in a published human dataset. Importantly, we directly validate gain-of-function (i.e., overexpressed) gene candidates highly ranked in both our *SB* screen as well as the formal rank aggregation of several independent genetic screens in a series of adoptive transfer experiments (4). These gene candidates (*AAK1*, *SPRR1B*, and *EHHADH*) are novel and should be considered as therapeutic targets in combination with ACT to improve T cell infiltration into solid tumors.

## Methods

### Mice

All mice were housed in specific pathogen-free facility at Mayo Clinic. The Mayo Clinic Animal Care and Use Committee (IACUC) approved all uses in this study. Mice were purchased from Jackson Laboratories (JAX stock #000664 and #003831).

### Overlap analysis

Peer-reviewed publications of *in vivo* mouse primary T cell genetic screens were manually identified and hand curated. The number of independent screens that identified each gene candidate was calculated and the full results appear in **Supplemental Table S1**. In the table, “NA” indicates that the gene was not tested in that screen, “k + 1” indicates that the gene was tested but not found to be a significant candidate by original authors, and fields containing numbers represent a significant candidate ranking (range 0-1) within the individual screen. Genetic drivers of T cell malignancy identified in a separate genetic screen (5) were filtered out of the final overlap list. Column I is the count of screens that identified each gene, and column J is the count of screens that tested each gene plus the Collier tumor driver screen. Column K is the percent of screens that tested each gene that identified it as a significant candidate.

### Rank aggregation

**Figure 1:**
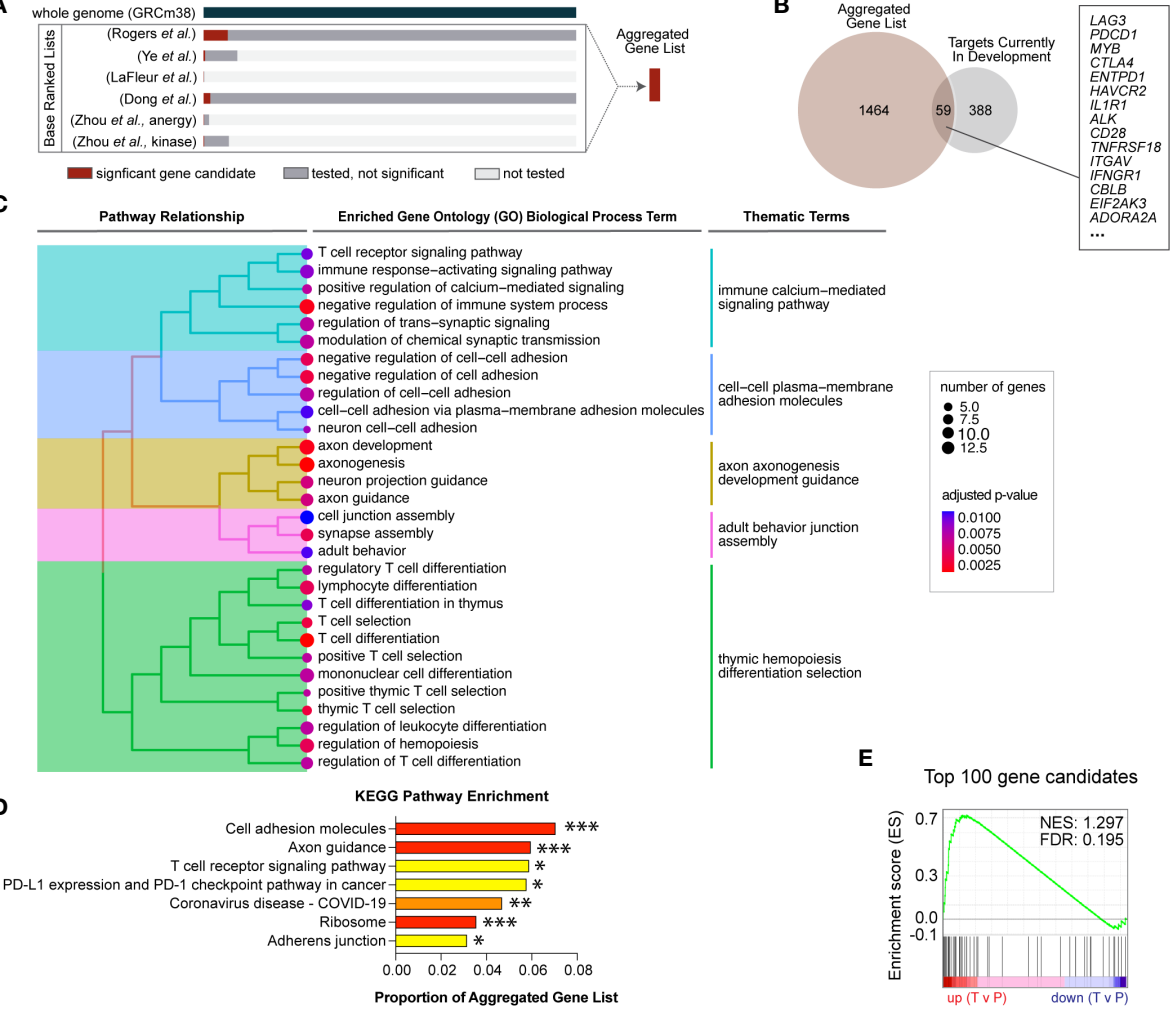
Novel gene candidates from five independent genetic screens were identified to regulate T cell infiltration into inflamed tissues. **(A)** Gene candidates from six independent screens were formally combined using a rank aggregation algorithm that accounts for variable genome coverage. The final aggregated list is ranked in descending order of selection strength (**Supplemental Table S2**). **(B)** Of the combined significant gene candidates, 59 are current targets for therapeutic development and the remaining 1,464 genes are novel therapeutic target candidates. **(C)** Aggregated gene candidates are significantly enriched in T cell signaling and cell adhesion functional annotations. Specific gene ontology (GO) terms with significant enrichment are displayed. Adjusted p-values are represented by dot color and the number of candidate genes represented in each GO term is represented by dot size. Enriched GO terms were subsequently grouped according to term similarity and gene overlap between terms. Groupings are depicted by the tree hierarchy structure and cluster themes. **(D)** Aggregated gene candidates are significantly enriched in KEGG pathways related to T cell activation and migration. Adjusted p-values are indicated (*** p < 0.005, ** p < 0.001, ** p < 0.05). **(E)** Gene set enrichment analysis (GSEA) revealed that the top 100 aggregated gene candidates are differentially expressed in human T cells located in peripheral blood (P) compared to intratumoral T cells (T) (FDR = 0.195).

Rank aggregation (RA) is a process of formally combining multiple ranked lists, or base rankers, into one ranked list, or aggregated ranker (6). Data from independent screens included a list of all gene candidates tested by screen and ranked on the significance measure chosen by original authors (e.g., p-value or FDR). Specifically, genes for which the significance measure was less than 0.05 were assigned a rank from 1 to k, and genes tested but not identified as significant were assigned the rank k + 1. These independent base rankers were then aggregated using the R package, ‘TopKLists’ (**Figure 1A**) using the Genome Reference Consortium Mouse Build 38 (GRCm38) genome as the underlying space. The rank aggregated output from the TopKLists geometric analysis is in **Supplemental Table S2**.

### Aggregated candidate pathway enrichment analyses

Overrepresentation analysis was performed on all rank aggregated gene candidates using g:Profiler2 v0.2.1 (7) and the gene ontology (GO) biological pathways (BP) (8) and Kyoto Encyclopedia of Genes and Genomes (KEGG) genes (9). The enrichment plot in **Figure 1C** was generated using GOSemSim v2.26.0 (10). Gene set enrichment analysis (GSEA) was performed using the top 100 rank aggregated gene candidates as the gene set and differential gene expression on tumor-infiltrating T cells versus peripheral T cells in treatment-naïve cancer patients from Zheng, et al. (11). Count data – including cluster, patient, source, and clonotype metadata – were downloaded from Zenodo (https://zenodo.org/record/5461803#.Y9fe4-xMH6Y). Original T cell cluster annotation was maintained, and analyses focused on 47 treatment-naive cancer patients from which both tumor and peripheral blood samples were taken. For each cluster, T cell data were bifurcated into two groups: tumor-infiltrating and non-infiltrating. Differential gene expression between tumor-infiltrating and non-infiltrating groups was determined using the Seurat (v4.3.0) FindMarkers function in R and ranked using the following equation: -log_10_(adjusted *P-*value)*(average log_2_FC). GSEA was performed against the top 100 ranked genes from our Aggregated Gene List using the GSEAPreranked tool (GSEA v4.1.0) from the Broad Institute (12).

### Tumor cell lines

Cell lines are tested annually for mycoplasma and were negative at time of last test. Cell lines were authenticated by STR analysis in 2018 (IDEXX BioResearch). EG7 cell lines were cultured in R10 medium (Gibco).

### *In vivo* adoptive T cell transfer

Full length *EHHADH*, *SPRR1B*, or truncated *AAK1* (*AAK1^ΔN125^*) cDNA was cloned into retroviral backbone MSCV (Takara) with a P2A-Thy1.1 reporter. An empty vector with an eGFP reporter was used as a control. Splenocytes from wild-type C57BL/6J mice were harvested, activated with Concanavalin A (2.5 µg/ml), and cultured in complete growth media supplemented with IL-2 (50 U/ml). HEK293T cells were transfected with pCL-ECO packaging vector and MSCV retroviral vector that overexpress the constructs indicated above. After two days in culture, the polyclonally activated splenocytes were transduced with the virus generated from the transfected HEK293T cells, and transduction efficiency were determined using Thy1.1 or eGFP reporter expression via flow cytometry. Tumor cells were resuspended in sterile PBS and 1x10^6^ EG7-OVA cells were injected subcutaneously into the rear flank(s) of 6 to 12-week-old mice. Tumor growth was monitored by caliper measurement and mice were euthanized before tumors reached 2 cm at largest diameter. Statistical methods were used to determine cohort size and researchers were not blinded. Seven days post tumor injection, 3.0x10^6^ transduced cells combined with 3.0x10^6^ empty vector with eGFP control were adoptively transferred intravenously.

### Flow cytometry to quantify intratumoral T cell infiltration

Tumors, spleens, or draining lymph nodes were excised and homogenized in RPMI using a gentleMACS dissociator (Miltenyi Biotec). Tumors were further processed with an enzymatic tumor dissociation kit (Miltenyi Biotec) followed by a mouse TIL positive selection kit (Miltenyi Biotec). Cell suspensions were passed through a 70 µm mesh filter and red blood cells were lysed with ACK buffer. Single cell suspensions were labeled with Ghost Red viability dye (Tonbo) and the following antibodies (BioLegend): TCRβ (H57-597), thy1.1 (OX-7), CD4 (GK1.5), CD8α (53-6.7), and Cxcr3 (CXCR3-173). Labeled cells were fixed and counted on an Attune NxT flow cytometer (Thermo Fisher). Flow data was analyzed using FlowJo software (**Supplemental Figure S1**). Experimental schematic was created with BioRender.

### Transwell migration assay

Splenocytes from WT C57BL/6J mice were transduced with either an empty vector with an eGFP reporter or AAK1^ΔN125^ with a Thy1.1 reporter. These cells were then placed on top of the porous membranes of Transwells with a 5.0 µm pores (Corning HTS Transwell), and the bottom of the wells contained either media only, CXCL9, or CXCL10 (Peprotech). After incubating for 90 minutes at 37°C, CountBright counting beads (Invitrogen) were added to each well, and the cells were stained for flow cytometry. Migration was analyzed based on the number of cells that migrated to the bottom of the wells compared to the total number of cells.

### Cytotoxicity and cytokine production assays

EG7-OVA viability upon co-culture with activated and genetically modified T cells was measured using two complementary assays. In the first assay, EG7-OVA cells, stained with CellTrace Violet (Invitrogen) were co-cultured with transduced T cells in a flat-bottom 96 well plate for an effector to target cell ratio of 25:1 and incubated at 37°C and 5% CO2 for 4 hours. Following co-culture, cells were labeled for flow cytometry with Ghost Red viability dye (Tonbo) and thy1.1 (OX-7) (BioLegend). In the second assay, EG7-OVA cells were seeded at 10,000 cells per well on flat-bottom 96-well plates coated with Fibronectin and incubated overnight at 37C. T cells, three days post transduction, were added to tumor cells at the indicated effector-to-target ratios followed by the addition of YOYO-3 Iodide (Invitrogen). YOYO-3 fluorescence was quantified with IncuCyte ZOOM (Essen BioScience) every 4 hours for 72 hours.

Cytokine production by T cells pre-treated for 5 hours with Cell Activation Cocktail (BioLegend) and Protei Cells were then labeled with Ghost Red viability dye (Tonbo) and the following antibodies (BioLegend): thy1.1 (OX-7), CD4 (GK1.5), CD8a (53-6.7), IL-10 (JES5-16E3), and IL-4 (11B11) and IFNγ (Invitrogen, XMG1.2). Labeled cells were then quantified using an Attune NxT flow cytometer (Thermo Fisher), and subsequent analysis were performed using FlowJo software.

### Statistical analysis

P values were calculated using the tests described in the individual figure legends using Graphpad Prism 9 (Graphpad Software) or R.

### Data sharing statement

All data and code are available online (https://github.com/RogersLabGroup/Rank-Aggregation).

## Results

### Overlap analysis identifies loss-of-function gene candidates identified by multiple screens

Unbiased and high-throughput forward genetic screens are instrumental in candidate identification, and we recently reported results from a genome-wide *Sleeping Beauty* (SB) screen to identify T cell genes regulating intratumoral T cell infiltration (4). To compare our results to similar screens, we identified five independent screens that interrogated T cell-intrinsic biology including activation, proliferation, and tissue infiltration (**Table 1**) (13–16). This large collection of complementary studies provides a unique opportunity to increase the strength of each underlying study by rigorously combining the results. A simple overlap between all six screens was calculated by counting the number of screens that identified each individual gene as a significant candidate. Gene candidates that were identified as driving oncogenes in T cell malignancies were removed (5). This yielded 27 genes that were identified in two or more independent screens including *Lag3, Dgkz*, and *Tnfrsf18* (**Supplemental Table S1**). All 27 are expected to be loss-of-function, as only the SB screen could produce gain-of-function mutations by inducing expression of full or truncated proteins. While these genes are likely to improve infiltration of adoptively transferred cells, the most strongly selected gain-of-function candidates uniquely identified in the SB screen were not considered using this overlap approach. Thus, we adopted a more rigorous approach to formally combine the independent screen results.

**Table 1:** Independent genetic screens to increase intratumoral T cell presence.

Screen Tool	Mutagenized Cell	Organism	Coverage	Method	Biology tested	Ref.
**shRNA**	Primary T cells (OT-1)	Mouse	248 T cell anergy/exhaustion genes and 1171 kinase/phosphatase genes	*In vivo* (ACT)	Antigen-specific T cell proliferation and intratumoral accumulation	(13)
**CRISPR**	Primary T cells (OT-1)	Mouse	Whole genome	*In vivo* (ACT)	Antigen-specific T cell proliferation and intratumoral accumulation	(14)
**CRISPR**	Primary T cells (P14 and OT-1)	Mouse	25 genes (TCR/cytokine signaling, costimulation, and metabolism)	*In vivo* (ACT)	Regulation of CD8+ T cell response to LCMV and MC38-Ova	(15)
**CRISPR**	Primary T cells (polyclonal)	Mouse	1,568 genes (membrane proteins)	*In vivo* (ACT)	T cell infiltration into glioblastoma	(16)
**SB**	Primary T cells (polyclonal)	Mouse	Whole genome	*In vivo* (endogenous)	T cell proliferation and intratumoral accumulation	(4)

### Rank aggregation highlights gain-of-function mutations as strongest candidates

Loss-of-function was tested in all screens, while gain-of-function (overexpression) mutations were tested in just one. Further, the coverage of each individual screen differed widely, ranging from 25 genes to whole genome, such that not all genes were tested in every screen (**Table 1**). To compensate for the differences in coverage of the underlying screens, a rank aggregation approach was adopted. Rank aggregation is the process of integrating several ranked base lists into a single aggregated ranking, weighting relative selection strength within the base list (**Figure 1A**). Based on a recent publication benchmarking algorithmic approaches for combining partial lists (e.g., 25 genes tested) with full lists (e.g., whole genome), the R package “TopKLists” with geometric output was chosen (6). The full, aggregated gene candidate list contains 1,523 genes, ranked in descending order of selection strength (**Table 2**, **Supplemental Table S2**).

**Table 2:** Top 15 ranked T cell gene candidates after screen aggregation.

Gene Symbol	Functional Description	Predicted LOF/GOF	Novel Target	# Screens Identified/# Tested
*AAK1*	Kinase that regulates CXCR3-mediated chemotaxis (4)	**GOF**	**✓**	1/3 (33%)
*LAG3*	T cell inhibitory coreceptor (17)	LOF		2/5 (40%)
*EHHADH*	Mitochondrial peroxisomal fatty acid metabolism enzyme (18)	**GOF**	**✓**	1/3 (33%)
*RGS16*	Expression inversely correlates with T cell migration after activation (19)	LOF	**✓**	1/4 (25%)
*MACROD2*	Epigenetic regulator frequently deactivated in ALL (20)	LOF	**✓**	1/2 (50%)
*CKB*	Kinase that enhances cytokine production and proliferation (21)	GOF	**✓**	1/3 (33%)
*DGKZ*	Kinase that limits T cell antitumor functions (22)	LOF		2/4 (50%)
*TOX*	Transcription factor that promotes T cell exhaustion (23)	LOF		1/3 (33%)
*NR4A2*	Transcription factor that limits T cell effector functions (24)	LOF		1/3 (33%)
*RNF214*	*Unknown*	GOF	**✓**	1/2 (50%)
*PDCD1*	Receptor important for maintaining peripheral T cell tolerance (25)	LOF		2/5 (40%)
*SPRR1B*	Promotes proliferation and migration (26), T cell function unknown	**GOF**	**✓**	1/2 (50%)
*PTPN2*	Phosphatase that promotes T cell dysfunction (27)	LOF		1/5 (20%)
*SON*	Splicing co-factor required for mitosis (28), T cell function unknown	GOF	**✓**	1/2 (50%)
*CCL4*	Chemokine that supports T cell adherence and migration (29)	LOF		1/3 (33%)

(GOF, gain-of-function mutation; LOF, loss-of-function mutation)

Shadings represent the three genes validated.

Cancer immunotherapy targets in all therapy categories, including ACT, and at all stages of development were exhaustively listed by the Cancer Research Institute and published in 2018 (30). To determine the number of novel candidate targets identified by genetic screens, the aggregated gene list was compared to the current immunotherapy targets list. Fifty-nine gene candidates were found to already be in therapeutic development (**Figure 1B**), while the remaining 1,464 represent novel candidate targets. Gene ontology enrichment analysis indicates that the novel candidates identified are significantly enriched for functions involved in T cell receptor (TCR) signaling, adhesion and migration, and T cell differentiation, indicating that activated T cell genes are well-represented (**Figure 1C**). Similarly, overrepresentation analysis with the Kyoto Encyclopedia of Genes and Genomes (KEGG) indicates the aggregated gene list is significantly enriched in T cell activation and adhesion processes (**Figure 1D**). These results are consistent with known biological processes important for T cell infiltration, supporting the validity of the genetic screen approaches.

The top 100 T cell gene candidates predicted to regulate T cell infiltration into inflamed tissues were used to define a custom “T cell infiltration” gene set for gene set enrichment analysis (GSEA). We hypothesized that the T cell infiltration gene set would be differentially expressed in intratumoral versus circulating T cells. T cell gene expression data from a publicly available human cancer patient dataset was used to compute T cell differential gene expression by location (11). Specifically, gene expression data were subsetted into tumor-infiltrating (T) or peripheral (P) locations, and differential expression was calculated for GSEA. Significantly differentially expressed genes by location were enriched for the T cell infiltration gene set (FDR <0.25) (**Figure 1E**). Enrichment varied by T cell subcluster, suggesting there may be opportunity to alter intratumoral accumulation of specific T cell subsets (**Supplemental Table S3**).

### Expressing gain-of-function candidates improves intratumoral accumulation in ACT

*AP2 associated kinase 1* (*Aak1*) was the strongest selected gene candidate in the SB screen, and Aak1 was subsequently observed to play a role in chemokine receptor endocytosis and T cell migration (4). Importantly, *Aak1* remained the strongest selected gene candidate even after integrating our screen results with additional independent screens and was chosen for experimental validation. In addition, two other strongly selected genes were chosen, *Sprr1b* and *Ehhadh*. All three were uniquely identified as gain-of-function (i.e., overexpression) mutations by our SB screen (4), and none of them are currently being considered for therapeutic development (**Table 2**).

**Figure 2:**
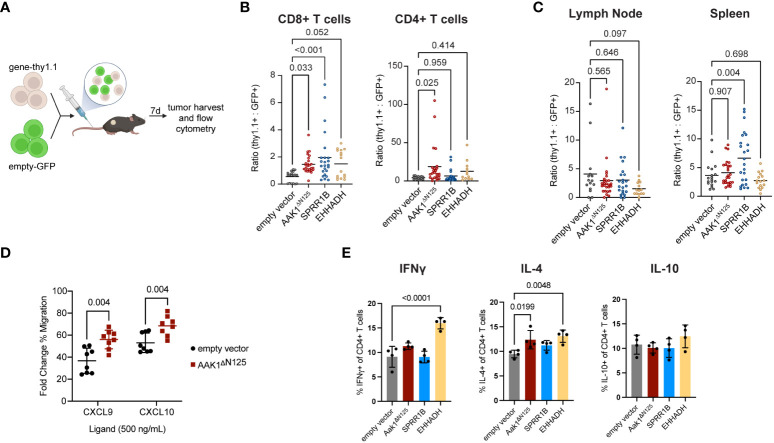
Infiltration of transduced T cells into solid tumors increased compared to empty vector control. **(A)** Experimental schematic of the adoptive transfer of cells transduced with empty vector (GFP) combined at 1:1 ratio with cells transduced with either empty, AAK1^ΔN125^, SPRR1B, or EHHADH vector (Thy1.1) into EG7 tumor-bearing mice. Schematic created with BioRender. **(B)** Ratio of cells expressing gene-of-interest to empty vector (thy1.1:GFP) of CD8^+^ and CD4^+^ T cell populations in tumors. P values were calculated using a one-way ANOVA with multiple comparison correction. **(C)** Ratio of TCRb+ cells expressing gene-of-interest to empty vector (thy1.1:GFP) within the draining lymph nodes and spleens. P values were calculated using a one-way ANOVA with multiple comparison correction. **(D)** *In vitro* transwell migration assay with primary murine T cells transduced with either empty vector (black circles) or Aak1^ΔN125^ (red squares) toward CXCR3 ligands CXCL9 and CXCL10. The y-axis reflects the ligand-induced % of migrated cells as a fold change over no-ligand baseline. P values were calculated using a two-sided *t* test. **(E)** *In vitro* cytokine production by CD4+ T cells after 24 hours co-culture with tumor cells. P values were calculated using a one-way ANOVA with multiple comparison correction.

The SB screen selected for overexpression of full-length *Sprr1b* and *Ehhadh* and truncated *Aak1* (*Aak1^ΔN125^*). Thus, polyclonally activated splenocytes from wild-type C57BL/6 mice stably transduced with either *AAK1* ^ΔN125^, *SPRR1B*, or *EHHADH* (P2A-Thy1.1 reporter) or with an empty vector (P2A-EGFP reporter) for a competitive infiltration experiment. After five days of expansion, genetically modified T cells were combined at a 1:1 ratio (gene of interest to empty vector control) and adoptively transferred into EG7-OVA tumor-bearing mice (tumor growth day seven). Seven days after adoptive transfer, gene-of-interest (thy1.1) and control (GFP) transduced T cells in tumors, tumor draining lymph nodes, and spleens were quantified by flow cytometry (**Figure 2A**).

*AAK1* ^ΔN125^ expression doubled the number of adoptively transferred T cells in tumors (both CD4^+^ and CD8^+^) while no significant differences in accumulation of adoptively transferred T cells were observed in the tumor draining lymph node or spleen (**Figures 2B, C**, **Supplementary Figure S2**). This suggests that *AAK1* ^ΔN125^ selectively augments T cell trafficking and infiltration into inflamed tissues, consistent with its role in promoting CXCR3-mediated chemotaxis (4). Indeed, *in vitro* T cell migration toward CXCR3 ligands CXCL9 and CXCL10 was enhanced upon *AAK1* ^ΔN125^ expression (**Figure 2D**). In contrast, *SPRR1B* significantly increased accumulation of adoptively transferred T cells in both the tumor (2.8-fold) and spleen (6.6-fold). The splenic increase may indicate a role in improving engraftment and/or *in vivo* proliferation of adoptively transferred cells consistent with the role of *SPRR1B* in supporting proliferation (26), though *in vitro* proliferation of *SPRR1B* expressing cells does not appear altered. Finally, *EHHADH* expression significantly increased accumulation of CD8+ T cells in tumors, but not CD4+ T cells, suggesting that *EHHADH* expression impacts T cell subsets differently.

In addition to efficient delivery of T cells to the solid tumor microenvironment, maintaining or augmenting other T cell functions, such as cytotoxicity and cytokine production, could enhance ACT efficacy. Importantly, cytotoxic capability of T cells expressing any of the three candidates was retained (**Supplementary Figure 4**). Expression of *EHHADH* resulted in increased production of IFNγ and IL-4 by CD4+ T cells, and expression of *AAK1^ΔN125^* modestly increased IL-4 production. Production of cytokines IFNγ, IL-4, and IL-10 by CD4+ T cells were unchanged by *SPRR1B* expression. Together, these results confirm that *AAK1* ^ΔN125^, *SPRR1B*, and *EHHADH* are good candidates to improve tissue infiltration of adoptively transferred cytotoxic T cells.

## Discussion

While ACT such as CAR-T cell immunotherapy have shown promising clinical results against liquid tumors, its therapeutic efficacy against solid tumors remains poor (31–33). Migrating T cells face numerous physical and biological barriers that prevent infiltration of tumors in addition to metabolic and physical barriers within the tumor microenvironment that prevent effective killing of tumor cells (31–33). Multiple generations of CAR-T constructs have been engineered with improvements meant to address unwanted effects such as cytokine release syndrome and overcome limitations such as persistence of the cells in patients with liquid cancers (34, 35). Additional innovations are needed to overcome limitations specific to solid tumors, like on-target/off-tumor killing and efficient delivery of therapeutic cells to the tumor. These can be addressed by intratumoral injection of immune cells; however, injections can be more invasive than intravenous delivery and regional delivery is less effective against metastases (36). Thus, identification of T cell intrinsic mechanisms governing this process could provide a unique avenue for therapeutic intervention.

The development of genetic tools such as RNAi, SB, and CRISPR has led to the advent of high-throughput screens, allowing for the identification of novel targets (37). Because these tools utilize different mechanisms of genetic manipulation, results using one technology may be used to preliminarily cross-validate results from another, thereby strengthening both sets of results. Thus, we established a manually curated list of independent datasets to combine based on cell type and the biology tested. Screens were required to have been performed in T cells, and all screens selected for efficient intratumoral infiltration regardless of screen tool used, thus it was deemed appropriate to combine results. Importantly, combining results from these complementary technologies collectively identified ~1500 T cell-intrinsic regulators of intratumoral T cell accumulation, 27 of which were identified as common targets by overlap analysis. Because the coverage ranged from a library of 25 genes to the whole genome, overlap analysis alone yields an incomplete picture, with many candidates not considered across all the screens. While every screen was equipped to identify loss-of-function mutations, the *SB* screen was also able to identify gain-of-function mutations (**Table 1**). Hypothesized gain-of-function mutations that were uniquely identified in the SB screen and that were highly ranked were not identified by loss-of-function screens. Thus, a more formal analysis was implemented.

Rank aggregation is an algorithm to combine screen results based on the relative ranks of novel candidates in the base screen and accounting for screen coverage (6). This ranking approach may be used to combine any independent screen data where similarities in screen design are shared. Aggregation of the six curated screens in **Table 1** resulted in a ranked list of all ~1500 gene candidates, with higher ranks associated with stronger genetic selection. Some of the genes identified are currently being targeted by immunotherapies of various developmental stages (e.g., *Pdcd1, Lag3*), but many represent truly innovative, non-immune checkpoint targets with great potential to synergize with immune checkpoint manipulations.

While pathway analyses did reveal significant enrichment in genes involved in TCR signaling (which includes some immune checkpoints), there was also substantial enrichment in T cell migration and adhesion genes that support trafficking into tumors (38). Further, the GSEA of the top 100 gene candidates shows enrichment in the tumor compared to peripheral blood from a human cancer patient dataset indicating accumulation within the tumor ( (11), **Figure 1E**). Together, these support the idea that the gene candidates identified may present novel therapeutic targets that can improve T cell infiltration of tumors and be complementary to immune checkpoint biology.

*Aak1* was the most strongly selected in both the SB screen as well as the aggregated gene list and is hypothesized to have a gain-of-function mutation ( (4), **Table 1**). *Aak1* is a kinase that promotes clathrin mediated endocytosis of multiple receptors and has been shown to modulate T cell trafficking *in vitro* migration toward chemokines CXCL9 and CXCL10 (4). The genetic selection for overexpression of a truncated mutant *Aak1* ^ΔN125^ suggested that loss of the N-terminus would promote *in vivo* infiltration. The truncation of amino acids 1-125 disrupts key residues previously shown to have kinase activity, which lead us to hypothesize that Aak1^ΔN125^ could also lack kinase activity and may function in a dominant-negative fashion (39), though additional experimental evidence is required.

*Sprr1B* and *Ehhadh* are two strongly selected gene candidates that are also overexpressed as full-length proteins. SPRR1B has been shown to play a role in cellular proliferation while EHHADH is an enzyme involved in the fatty acid β-oxidation pathway (26, 40). *SPRR1B* may be oncogenic in epithelial cells, while *EHHADH* has also been linked with non-lymphoid cancers (41–44). Mechanistically, overexpression of *Sprr1b* in human oral squamous cell carcinoma cell lines has been found to impact MAP kinase signaling (45) but none of these candidates have been studied in T cells. Importantly, overexpression of all three chosen gene candidates – *AAK1* ^ΔN125^, *SPRR1B*, and *EHHADH* – led to increased infiltration of transduced T cells, validating these as novel candidates.

The *in vivo* validation performed here utilized polyclonally activated cells. In the future, the candidates validated here may be combined with a tumor-specific engineered receptor (CAR or TCR) to increase delivery of therapeutic cells to the tumor microenvironment. There were interesting and potentially important differences in infiltration observed between the three candidates. *AAK1* ^ΔN125^ increased infiltration of both CD4+ and CD8+ T cells, while *SPRR1B* and *EHHADH* increased the infiltration of only CD8+ T cells (**Figure 2A**). The presence of CD8+ tumor infiltrating lymphocytes (TILs) has been shown to have better tumor killing and has been associated with better clinical prognosis (46, 47). In contrast, the presence of CD4+ TILs is associated with both positive and negative prognosis and tumor killing depending on tumor type, but overall may be beneficial to anti-tumor efficacy (47–49). This knowledge could be beneficial in choosing which candidate is appropriate for the desired outcome. For example, CD4+ CAR-T cells were better than CD8+ CAR-T cells at controlling glioblastoma growth (50). Further, CD4+ T cells have been shown to help alleviate exhaustion of CD8+ T cells during chronic infection, and CD4+ T cells have been shown to have longer persistence and better potency in glioblastoma (50, 51), so *AAK1* ^ΔN125^ expression in these settings could be desirable. Alternately, CD4+ CAR-T cells can exacerbate cytokine release syndrome in liquid tumors (52). If the same is true for solid tumors, *SPRR1B* or *EHHADH* may be better choices. Further, interferon-gamma (IFNγ) production has been associated with CD4 CAR T-cell tumor killing and increased patient survival, and IL-4 production by CD4+ T cells has been shown to impact tumor growth, which would support *EHHADH* as a good candidate [(53, 54), **Figure 2E**].

Engraftment and persistence of adoptively transferred cells also present a major challenge. While the experimental approach adopted in this paper does not directly address engraftment or persistence longer than seven days, it is interesting to note that *SPRR1B* expressing cells, but not *AAK1* ^ΔN125^ or *EHHADH*, were also significantly enriched in the spleen. While SPRR1B may improve overall engraftment and infiltration, the infiltration does not appear to be tumor specific, which may decrease translational interest in this gene candidate. We hypothesize that this could result from enhanced engraftment and aim to test this directly in future work.

Overexpression of these three candidates could also impact an endogenous T cell response at earlier steps than tumor infiltration (e.g., T cell priming), which were captured by the original screen design but not evaluated in this experiment. A role in promoting T cell priming is less relevant for ACT, where T cells are stimulated *ex vivo* prior to genetic modification. However, efficient priming is critical in therapies boosting endogenous T cell reactions like immune checkpoint blockade, and future experiments exploring the impact of these gene candidates on additional T cell processes will be important.

In summary, forward genetic screens are an efficient approach to identify truly innovative gene candidates without *a priori* knowledge of the importance of different biological pathways. We and others have performed screens in T cells to identify drivers of T cell infiltration into solid tumors using a variety of technical approaches. These collective data can be used to strengthen observations in each individual dataset, but the method of combining datasets must be carefully considered. Rank aggregation was chosen as the best method to rigorously combine candidates from independent screens. Finally, three of three novel candidates chosen for validation were successful in improving infiltration of adoptively transferred cells, though the mechanisms by which they do so are likely different based on the observed differences in CD4+/CD8+ subsets and tissue distribution post transfer. This study provides rationale for investigating all top gene candidates, and especially *AAK1* ^ΔN125^ *, EHHADH*, and *SPRR1B*, for therapeutic consideration in cancer immunotherapy.

## Data availability statement

The original contributions presented in the study are included in the article/**Supplementary Material**. Further inquiries can be directed to the corresponding author.

## Ethics statement

Ethical approval was not required for the study involving humans in accordance with the local legislation and institutional requirements. Written informed consent to participate in this study was not required from the participants or the participants’ legal guardians/next of kin in accordance with the national legislation and the institutional requirements. The animal study was approved by Mayo Clinic Animal Care and Use Committee (IACUC). The study was conducted in accordance with the local legislation and institutional requirements.

## Author contributions

VVV: curation and pre-processing of screen results, experimental design, data acquisition and analysis, writing – original draft. RMH: data acquisition and analysis, writing – original draft. IG: curation and preprocessing of screen results, data acquisition assistance, writing – review and editing. GJJ: generated and validated key reagents, writing – review and editing. LMR: conceptualization, funding acquisition, project administration and supervision, writing – review and editing. All authors contributed to the article and approved the submitted version.

## Supplementary material

Supplementary Figure 1 Representative gating strategy of intratumoral T cell populations.

Supplementary Figure 2 Adoptively transferred T cells are also detectable in spleen and tumor draining lymph node at time of tumor harvest. **(A),** Frequency of bulk intratumoral TCRb+ cells (of live/singlets) expressing empty vector control (GFP) or gene-of-interest with fold-increase (thy1.1/GFP) as indicated. *AAK1* ^ΔN125^ (*, P=0.0234) and *SPRR1B* (**, P=0.0014) induced significantly more overall infiltration than empty vector. Bulk T cell infiltration of cells expressing *EHHADH* did not achieve significance (P=0.2310), however, considering the CD4/CD8 subsets separately revealed that increased infiltration was limited to the CD8+ subset. **(B),** Frequency of bulk TCRb+ cells expressing empty vector control (GFP) or gene-of-interest in tumor draining lymph nodes with fold-increase (thy1.1/GFP) as indicated (none are significant). **(C),** Frequency of infiltrating bulk TCRb+ cells expressing empty vector control (GFP) or gene-of-interest in spleens with fold fold-increase (thy1.1/GFP) as indicated (*SPRR1B* p = 0.0041).

Supplementary Figure 3
*In vitro* proliferation of transduced T cells (empty vector, *AAK1* ^ΔN125^, *SPRR1B*). No proliferation advantage was determined upon transduction with *AAK1* ^ΔN125^ or *SPRR1B* compared to cells transduced with empty vector.

Supplementary Figure 4 No significant differences were shown in two separate *in vitro* cytotoxicity assays among transduced T cells co-cultured with EG7 cells. **(A)**. *In vitro* LDH cytotoxicity assay of transduced T cells (empty vector, *AAK1* ^ΔN125^, *SPRR1B*, *EHHADH)* co-cultured with EG7 cells for four hours. **(B)**. *In vitro* cytotoxicity assay of transduced T cells (empty vector, *AAK1* ^ΔN125^, *SPRR1B*, *EHHADH)* co-cultured with EG7 cells using YOYO-3 Iodide and visualized using IncuCyte ZOOM over 72 hours. **(C)**. YOYO-3 Iodide expression at different effector-to-target (E:T) ratios after 72-hour incubation. **(D)**. YOYO-3 Iodide expression at different effector-to-target (E:T) ratios after 72 hours normalized with time 0 (YOYO-3 Iodide T72/YOYO-3 Iodide T0).

Supplementary Table 1 Overlap analysis for all gene candidates including percentage of screens identifying the indicated gene of all screens that tested that gene.

Supplementary Table 2 The rank aggregated output from the TopKLists geometric analysis in descending order of selection strength.

Supplementary Table 3 GSEA results by T cell cluster.

## References

[1] Kershaw MH Westwood JA Darcy PK. Gene-engineered t cells for cancer therapy. Nat Rev Cancer (2013) 13(8):525–41. doi: 10.1038/nrc3565 23880905

[2] Xin Yu J Hubbard-Lucey VM Tang J. The global pipeline of cell therapies for cancer. Nat Rev Drug Discovery (2019) 18(11):821–2. doi: 10.1038/d41573-019-00090-z 31673124

[3] Morotti M Albukhari A Alsaadi A Artibani M Brenton JD Curbishley SM. Promises and challenges of adoptive t-cell therapies for solid tumours. Br J Cancer (2021) 124(11):1759–76. doi: 10.1038/s41416-021-01353-6 PMC8144577 33782566

[4] Rogers LM Wang Z Mott SL Dupuy AJ Weiner GJ. A genetic screen to identify gain- and loss-of-Function modifications that enhance t-cell infiltration into tumors. Cancer Immunol Res (2020) 8(9):1206–14. doi: 10.1158/2326-6066.CIR-20-0056 PMC7483799 32611665

[5] Collier LS Adams DJ Hackett CS Bendzick LE Akagi K Davies MN. Whole-body sleeping beauty mutagenesis can cause penetrant Leukemia/Lymphoma and rare high-grade glioma without associated embryonic lethality. Cancer Res (2009) 69(21):8429–37. doi: 10.1158/0008-5472.CAN-09-1760 PMC2771123 19843846

[6] Li X Wang X Xiao G. A comparative study of rank aggregation methods for partial and top ranked lists in genomic applications. Brief Bioinform (2019) 20(1):178–89. doi: 10.1093/bib/bbx101 PMC6357556 28968705

[7] Raudvere U Kolberg L Kuzmin I Arak T Adler P Peterson H. G:Profiler: A web server for functional enrichment analysis and conversions of gene lists (2019 update). Nucleic Acids Res (2019) 47(W1):W191–W8. doi: 10.1093/nar/gkz369 PMC6602461 31066453

[8] Gene Ontology C. The gene ontology resource: Enriching a gold mine. Nucleic Acids Res (2021) 49(D1):D325–D34. doi: 10.1093/nar/gkaa1113 PMC7779012 33290552

[9] Kanehisa M Goto S. Kegg: Kyoto encyclopedia of genes and genomes. Nucleic Acids Res (2000) 28(1):27–30. doi: 10.1093/nar/28.1.27 10592173 PMC102409

[10] Yu G. Gene ontology semantic similarity analysis using gosemsim. Methods Mol Biol (2020) 2117:207–15. doi: 10.1007/978-1-0716-0301-7_11 31960380

[11] Zheng L Qin S Si W Wang A Xing B Gao R. Pan-cancer single-cell landscape of tumor-infiltrating t cells. Science (2021) 374(6574):abe6474. doi: 10.1126/science.abe6474 34914499

[12] Subramanian A Tamayo P Mootha VK Mukherjee S Ebert BL Gillette MA. Gene set enrichment analysis: A knowledge-based approach for interpreting genome-wide expression profiles. Proc Natl Acad Sci USA (2005) 102(43):15545–50. doi: 10.1073/pnas.0506580102 PMC1239896 16199517

[13] Zhou P Shaffer DR Alvarez Arias DA Nakazaki Y Pos W Torres AJ. *In vivo* discovery of immunotherapy targets in the tumour microenvironment. Nature (2014) 506(7486):52–7. doi: 10.1038/nature12988 PMC4052214 24476824

[14] Dong MB Wang G Chow RD Ye L Zhu L Dai X. Systematic immunotherapy target discovery using genome-scale in vivo crispr screens in Cd8 t cells. Cell (2019) 178(5):1189–204 e23. doi: 10.1016/j.cell.2019.07.044 31442407 PMC6719679

[15] LaFleur MW Nguyen TH Coxe MA Yates KB Trombley JD Weiss SA. A crispr-Cas9 delivery system for in vivo screening of genes in the immune system. Nat Commun (2019) 10(1):1668. doi: 10.1038/s41467-019-09656-2 30971695 PMC6458184

[16] Ye L Park JJ Dong MB Yang Q Chow RD Peng L. *In vivo* crispr screening in Cd8 t cells with aav-sleeping beauty hybrid vectors identifies membrane targets for improving immunotherapy for glioblastoma. Nat Biotechnol (2019) 37(11):1302–13. doi: 10.1038/s41587-019-0246-4 PMC6834896 31548728

[17] Maruhashi T Sugiura D Okazaki IM Okazaki T. Lag-3: From molecular functions to clinical applications. J Immunother Cancer (2020) 8(2). doi: 10.1136/jitc-2020-001014 PMC7488795 32929051

[18] Houten SM Denis S Argmann CA Jia Y Ferdinandusse S Reddy JK. Peroxisomal l-bifunctional enzyme (Ehhadh) is essential for the production of medium-chain dicarboxylic acids. J Lipid Res (2012) 53(7):1296–303. doi: 10.1194/jlr.M024463 PMC3371241 22534643

[19] Agenes F Bosco N Mascarell L Fritah S Ceredig R. Differential expression of regulator of g-protein signalling transcripts and in vivo migration of Cd4+ naive and regulatory t cells. Immunology (2005) 115(2):179–88. doi: 10.1111/j.1365-2567.2005.02146.x PMC1782143 15885123

[20] Chen C Bartenhagen C Gombert M Okpanyi V Binder V Rottgers S. Next-Generation-Sequencing of recurrent childhood high hyperdiploid acute lymphoblastic leukemia reveals mutations typically associated with high risk patients. Leuk Res (2015) 39(9):990–1001. doi: 10.1016/j.leukres.2015.06.005 26189108

[21] Zhang Y Li H Wang X Gao X Liu X. Regulation of t cell development and activation by creatine kinase b. PloS One (2009) 4(4):e5000. doi: 10.1371/journal.pone.0005000 19337362 PMC2659424

[22] Jung IY Kim YY Yu HS Lee M Kim S Lee J. Crispr/Cas9-mediated knockout of dgk improves antitumor activities of human t cells. Cancer Res (2018) 78(16):4692–703. doi: 10.1158/0008-5472.CAN-18-0030 29967261

[23] Liang C Huang S Zhao Y Chen S Li Y. Tox as a potential target for immunotherapy in lymphocytic malignancies. biomark Res (2021) 9(1):20. doi: 10.1186/s40364-021-00275-y 33743809 PMC7981945

[24] Chen J Lopez-Moyado IF Seo H Lio CJ Hempleman LJ Sekiya T. Nr4a transcription factors limit car t cell function in solid tumours. Nature (2019) 567(7749):530–4. doi: 10.1038/s41586-019-0985-x PMC6546093 30814732

[25] Wei SC Duffy CR Allison JP. Fundamental mechanisms of immune checkpoint blockade therapy. Cancer Discovery (2018) 8(9):1069–86. doi: 10.1158/2159-8290.CD-18-0367 30115704

[26] Zhang Z Shi R Xu S Li Y Zhang H Liu M. Identification of small proline-rich protein 1b (Sprr1b) as a prognostically predictive biomarker for lung adenocarcinoma by integrative bioinformatic analysis. Thorac Cancer (2021) 12(6):796–806. doi: 10.1111/1759-7714.13836 33501784 PMC7952803

[27] LaFleur MW Nguyen TH Coxe MA Miller BC Yates KB Gillis JE. Ptpn2 regulates the generation of exhausted Cd8(+) t cell subpopulations and restrains tumor immunity. Nat Immunol (2019) 20(10):1335–47. doi: 10.1038/s41590-019-0480-4 PMC6754306 31527834

[28] Huen MS Sy SM Leung KM Ching YP Tipoe GL Man C. Son is a spliceosome-associated factor required for mitotic progression. Cell Cycle (2010) 9(13):2679–85. doi: 10.4161/cc.9.13.12151 PMC3040851 20581448

[29] Quandt J Dorovini-Zis K. The Beta chemokines Ccl4 and Ccl5 enhance adhesion of specific Cd4+ T cell subsets to human brain endothelial cells. J Neuropathol Exp Neurol (2004) 63(4):350–62. doi: 10.1093/jnen/63.4.350 15099025

[30] Tang J Pearce L O'Donnell-Tormey J Hubbard-Lucey VM. Trends in the global immuno-oncology landscape. Nat Rev Drug Discovery (2018) 17(12):922. doi: 10.1038/nrd.2018.202 30361553

[31] Melssen MM Sheybani ND Leick KM Slingluff CL Jr.. Barriers to immune cell infiltration in tumors. J Immunother Cancer (2023) 11(4). doi: 10.1136/jitc-2022-006401 PMC10124321 37072352

[32] D'Aloia MM Zizzari IG Sacchetti B Pierelli L Alimandi M. Car-t cells: The long and winding road to solid tumors. Cell Death Dis (2018) 9(3):282. doi: 10.1038/s41419-018-0278-6 29449531 PMC5833816

[33] Foeng J Comerford I McColl SR. Harnessing the chemokine system to home car-t cells into solid tumors. Cell Rep Med (2022) 3(3):100543. doi: 10.1016/j.xcrm.2022.100543 35492880 PMC9040186

[34] Tomasik J Jasinski M Basak GW. Next generations of car-t cells - new therapeutic opportunities in hematology? Front Immunol (2022) 13:1034707. doi: 10.3389/fimmu.2022.1034707 36389658 PMC9650233

[35] Sterner RC Sterner RM. Car-t cell therapy: Current limitations and potential strategies. Blood Cancer J (2021) 11(4):69. doi: 10.1038/s41408-021-00459-7 33824268 PMC8024391

[36] Sridhar P Petrocca F. Regional delivery of chimeric antigen receptor (Car) t-cells for cancer therapy. Cancers (Basel) (2017) 9(7). doi: 10.3390/cancers9070092 PMC5532628 28718815

[37] Schuster A Erasimus H Fritah S Nazarov PV van Dyck E Niclou SP. Rnai/Crispr screens: From a pool to a valid hit. Trends Biotechnol (2019) 37(1):38–55. doi: 10.1016/j.tibtech.2018.08.002 30177380

[38] Harjunpaa H Llort Asens M Guenther C Fagerholm SC. Cell adhesion molecules and their roles and regulation in the immune and tumor microenvironment. Front Immunol (2019) 10:1078. doi: 10.3389/fimmu.2019.01078 31231358 PMC6558418

[39] Conner SD Schmid SL. Differential requirements for ap-2 in clathrin-mediated endocytosis. J Cell Biol (2003) 162(5):773–9. doi: 10.1083/jcb.200304069 PMC2172816 12952931

[40] Zhou T Cao L Du Y Qin L Lu Y Zhang Q. Gypenosides ameliorate high-fat diet-induced nonalcoholic fatty liver disease in mice by regulating lipid metabolism. PeerJ (2023) 11:e15225. doi: 10.7717/peerj.15225 37065701 PMC10103699

[41] de Lanna CA da Silva BNM de Melo AC Bonamino MH Alves LDB Pinto LFR. Oral lichen planus and oral squamous cell carcinoma share key oncogenic signatures. Sci Rep (2022) 12(1):20645. doi: 10.1038/s41598-022-24801-6 36450755 PMC9712651

[42] Njoku K Pierce A Geary B Campbell AE Kelsall J Reed R. Quantitative swath-based proteomic profiling of urine for the identification of endometrial cancer biomarkers in symptomatic women. Br J Cancer (2023) 128(9):1723–32. doi: 10.1038/s41416-022-02139-0 PMC10133303 36807337

[43] Jiang W Zhang L Guo Q Wang H Ma M Sun J. Identification of the pathogenic biomarkers for hepatocellular carcinoma based on rna-seq analyses. Pathol Oncol Res (2019) 25(3):1207–13. doi: 10.1007/s12253-019-00596-2 30680535

[44] Okamura S Yoshino H Kuroshima K Tsuruda M Osako Y Sakaguchi T. Ehhadh contributes to cisplatin resistance through regulation by tumor-suppressive micrornas in bladder cancer. BMC Cancer (2021) 21(1):48. doi: 10.1186/s12885-020-07717-0 33430801 PMC7798329

[45] Michifuri Y Hirohashi Y Torigoe T Miyazaki A Fujino J Tamura Y. Small proline-rich protein-1b is overexpressed in human oral squamous cell cancer stem-like cells and is related to their growth through activation of map kinase signal. Biochem Biophys Res Commun (2013) 439(1):96–102. doi: 10.1016/j.bbrc.2013.08.021 23954638

[46] Li F Li C Cai X Xie Z Zhou L Cheng B. The association between Cd8+ tumor-infiltrating lymphocytes and the clinical outcome of cancer immunotherapy: A systematic review and meta-analysis. EClinicalMedicine (2021) 41:101134. doi: 10.1016/j.eclinm.2021.101134 34585125 PMC8452798

[47] Schulze AB Evers G Gorlich D Mohr M Marra A Hillejan L. Tumor infiltrating t cells influence prognosis in stage i-iii non-small cell lung cancer. J Thorac Dis (2020) 12(5):1824–42. doi: 10.21037/jtd-19-3414a PMC7330340 32642087

[48] Wong SB Bos R Sherman LA. Tumor-specific Cd4+ t cells render the tumor environment permissive for infiltration by low-avidity Cd8+ t cells. J Immunol (2008) 180(5):3122–31. doi: 10.4049/jimmunol.180.5.3122 18292535

[49] Wang LX Shu S Disis ML Plautz GE. Adoptive transfer of tumor-primed, in vitro-activated, Cd4+ t effector cells (Tes) combined with Cd8+ tes provides intratumoral te proliferation and synergistic antitumor response. Blood (2007) 109(11):4865–76. doi: 10.1182/blood-2006-09-045245 PMC1885514 17284532

[50] Wang D Aguilar B Starr R Alizadeh D Brito A Sarkissian A. Glioblastoma-targeted Cd4+ car t cells mediate superior antitumor activity. JCI Insight (2018) 3(10). doi: 10.1172/jci.insight.99048 PMC6012522 29769444

[51] Lu YJ Barreira-Silva P Boyce S Powers J Cavallo K Behar SM. Cd4 t cell help prevents Cd8 t cell exhaustion and promotes control of mycobacterium tuberculosis infection. Cell Rep (2021) 36(11):109696. doi: 10.1016/j.celrep.2021.109696 34525366 PMC8466141

[52] Bove C Arcangeli S Falcone L Camisa B El Khoury R Greco B. Cd4 car-t cells targeting Cd19 play a key role in exacerbating cytokine release syndrome, while maintaining long-term responses. J Immunother Cancer (2023) 11(1). doi: 10.1136/jitc-2022-005878 PMC9809278 36593069

[53] Boulch M Cazaux M Cuffel A Guerin MV Garcia Z Alonso R. Tumor-intrinsic sensitivity to the pro-apoptotic effects of ifn-gamma is a major determinant of Cd4(+) car t-cell antitumor activity. Nat Cancer (2023) 4(7):968–83. doi: 10.1038/s43018-023-00570-7 PMC10368531 37248395

[54] Li S Liu M Do MH Chou C Stamatiades EG Nixon BG. Cancer immunotherapy *Via* targeted tgf-beta signalling blockade in T(H) cells. Nature (2020) 587(7832):121–5. doi: 10.1038/s41586-020-2850-3 PMC8353603 33087933

