# Supplementary figures and images for: Rank aggregation of independent genetic screen results highlights new strategies for adoptive cellular transfer therapy of cancer
Source: Front Immunol. 2023 Dec 8;14:1235131. doi: 10.3389/fimmu.2023.1235131 (PMC10748423; doi:10.3389/fimmu.2023.1235131)

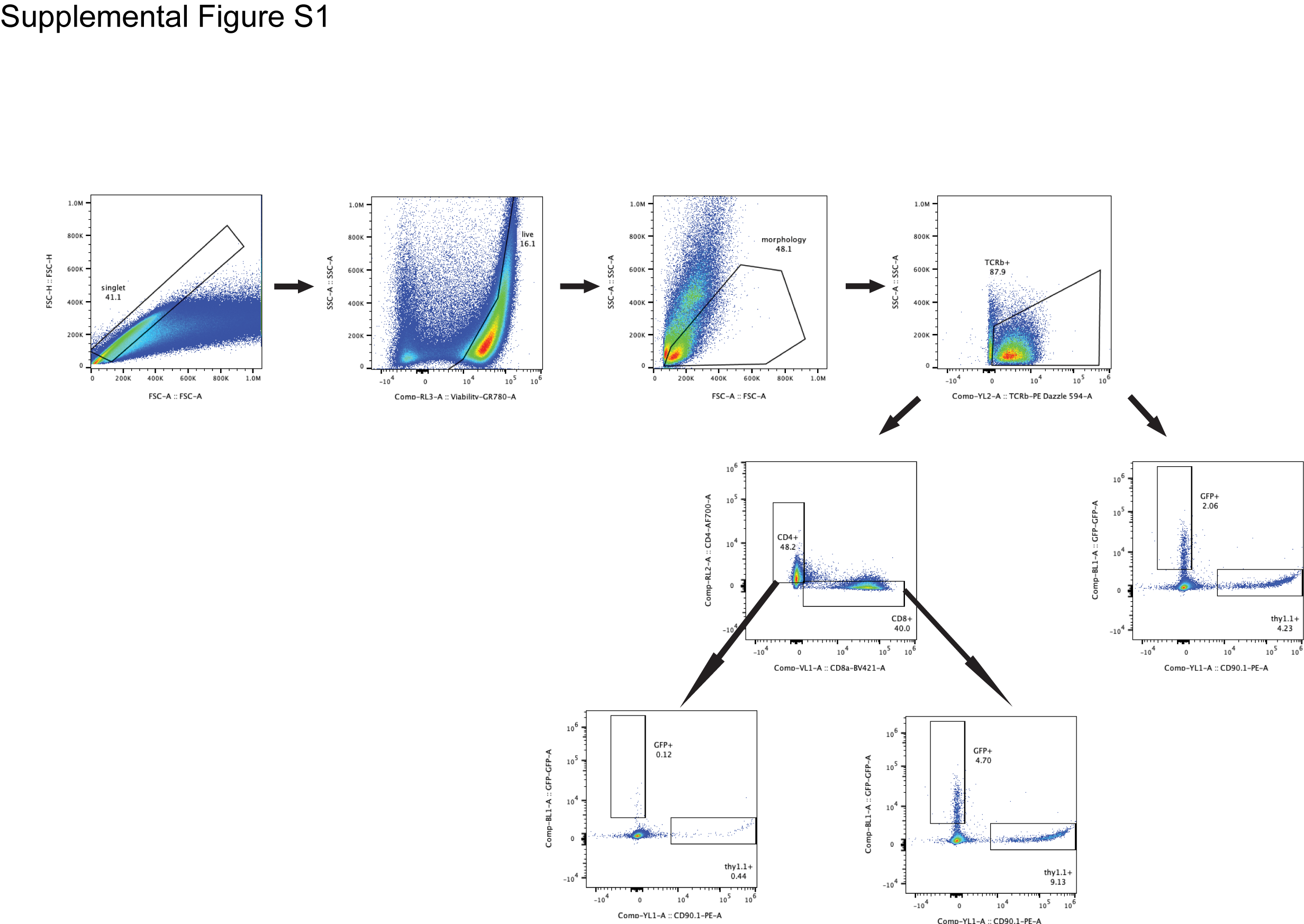

Supplement: Supplementary Figure 1 — Representative gating strategy of intratumoral T cell populations. [file Image_1.tif]

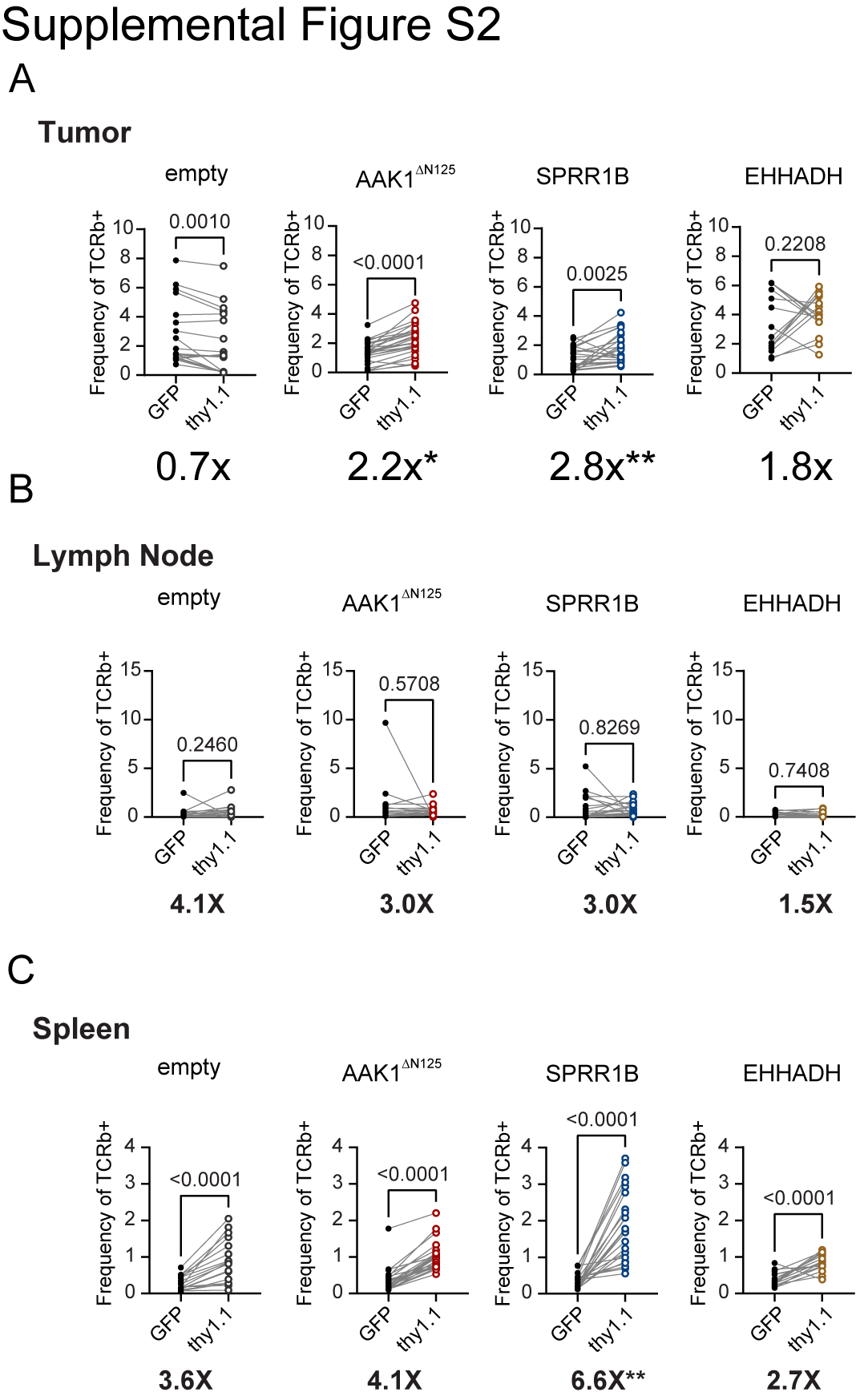

Supplement: Supplementary Figure 2 — Adoptively transferred T cells are also detectable in spleen and tumor draining lymph node at time of tumor harvest. (A), Frequency of bulk intratumoral TCRb+ cells (of live/singlets) expressing empty vector control (GFP) or gene-of-interest with fold-increase (thy1.1/GFP) as indicated. AAK1 ΔN125 (*, P=0.0234) and SPRR1B (**, P=0.0014) induced significantly more overall infiltration than empty vector. Bulk T cell infiltration of cells expressing EHHADH did not achieve significance (P=0.2310), however, considering the CD4/CD8 subsets separately revealed that increased infiltration was limited to the CD8+ subset. (B), Frequency of bulk TCRb+ cells expressing empty vector control (GFP) or gene-of-interest in tumor draining lymph nodes with fold-increase (thy1.1/GFP) as indicated (none are significant). (C), Frequency of infiltrating bulk TCRb+ cells expressing empty vector control (GFP) or gene-of-interest in spleens with fold fold-increase (thy1.1/GFP) as indicated (SPRR1B p = 0.0041). [file Image_2.tif]

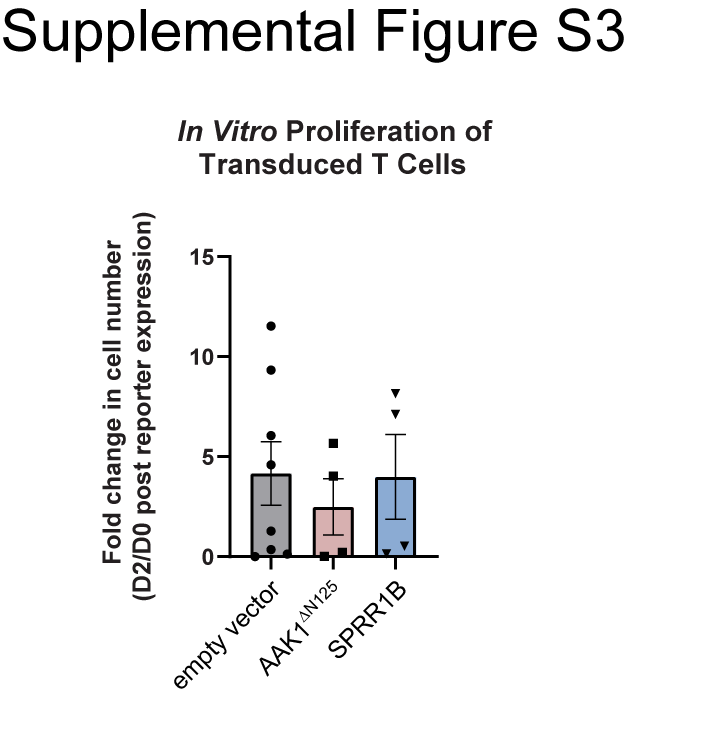

Supplement: Supplementary Figure 3 — In vitro proliferation of transduced T cells (empty vector, AAK1 ΔN125, SPRR1B). No proliferation advantage was determined upon transduction with AAK1 ΔN125 or SPRR1B compared to cells transduced with empty vector. [file Image_3.tif]

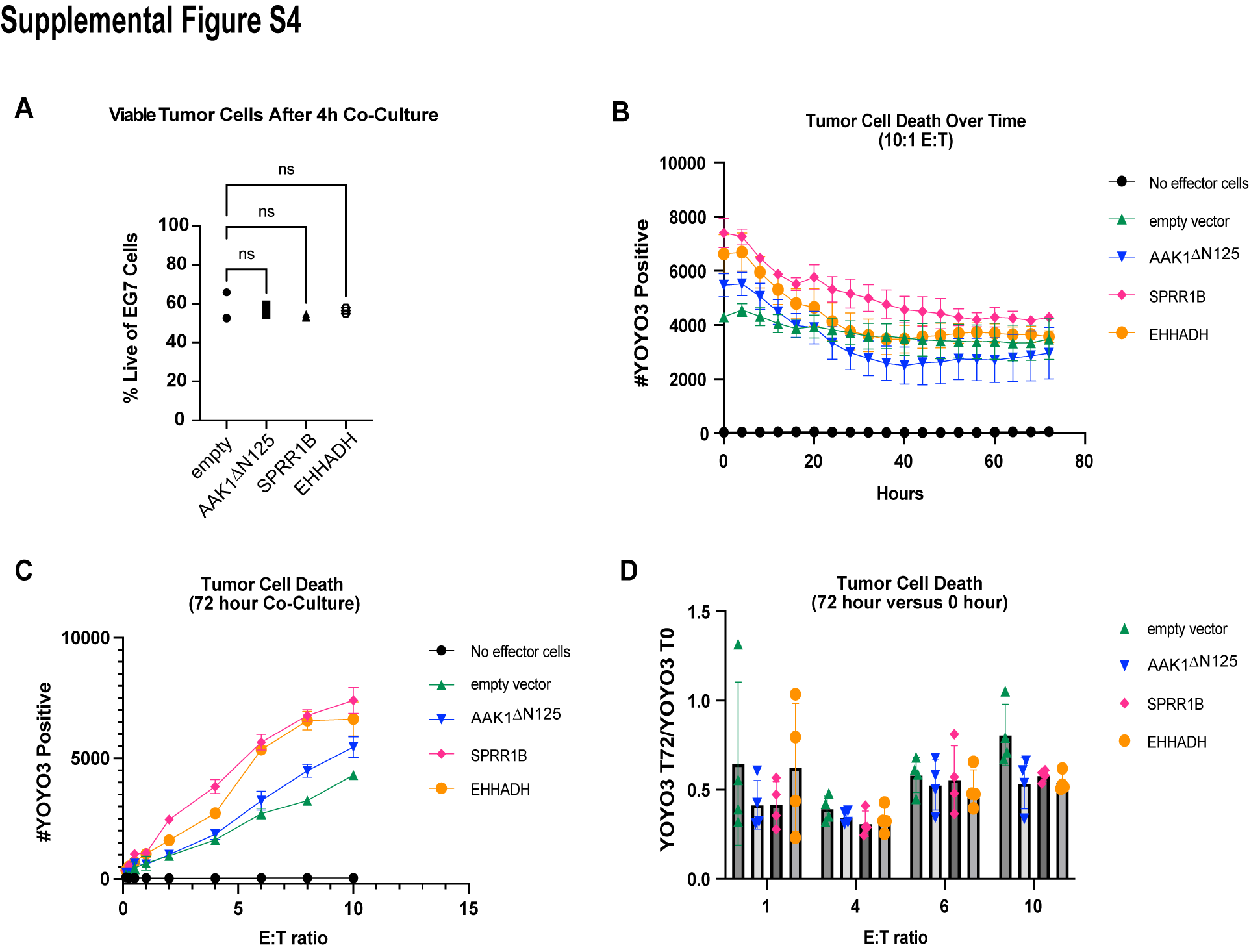

Supplement: Supplementary Figure 4 — No significant differences were shown in two separate in vitro cytotoxicity assays among transduced T cells co-cultured with EG7 cells. (A). In vitro LDH cytotoxicity assay of transduced T cells (empty vector, AAK1 ΔN125, SPRR1B, EHHADH) co-cultured with EG7 cells for four hours. (B). In vitro cytotoxicity assay of transduced T cells (empty vector, AAK1 ΔN125, SPRR1B, EHHADH) co-cultured with EG7 cells using YOYO-3 Iodide and visualized using IncuCyte ZOOM over 72 hours. (C). YOYO-3 Iodide expression at different effector-to-target (E:T) ratios after 72-hour incubation. (D). YOYO-3 Iodide expression at different effector-to-target (E:T) ratios after 72 hours normalized with time 0 (YOYO-3 Iodide T72/YOYO-3 Iodide T0). [file Image_4.tif]
